# Immunomodulatory effects of different intravenous immunoglobulin preparations in chronic lymphocytic leukemia

**DOI:** 10.1038/s41598-021-92412-8

**Published:** 2021-06-21

**Authors:** Ana Colado, Esteban Enrique Elías, Valeria Judith Sarapura Martínez, Gregorio Cordini, Pablo Morande, Fernando Bezares, Mirta Giordano, Romina Gamberale, Mercedes Borge

**Affiliations:** 1 Laboratorio de Inmunología Oncológica, Instituto de Medicina Experimental (IMEX), CONICET-Academia Nacional de Medicina (ANM), Pacheco de Melo 3081 (1425), CABA, Argentina; 2 Tumor Stroma Interactions, Department of Oncology, Luxembourg Institute of Health, 1526 Luxembourg, Luxembourg; 3 Hospital General de Agudos Dr. Teodoro Álvarez, CABA, Argentina; 4 Departamento de Microbiología, Parasitología e Inmunología, Facultad de Medicina, Universidad de Buenos Aires, CABA, Argentina

**Keywords:** Chronic lymphocytic leukaemia, Lymphocytes

## Abstract

Hypogammaglobulinemia is the most frequently observed immune defect in chronic lymphocytic leukemia (CLL). Although CLL patients usually have low serum levels of all isotypes (IgG, IgM and IgA), standard immunoglobulin (Ig) preparations for replacement therapy administrated to these patients contain more than 95% of IgG. Pentaglobin is an Ig preparation of intravenous application (IVIg) enriched with IgM and IgA (IVIgGMA), with the potential benefit to restore the Ig levels of all isotypes. Because IVIg preparations at high doses have well-documented anti-inflammatory and immunomodulatory effects, we aimed to evaluate the capacity of Pentaglobin and a standard IVIg preparation to affect leukemic and T cells from CLL patients. In contrast to standard IVIg, we found that IVIgGMA did not modify T cell activation and had a lower inhibitory effect on T cell proliferation. Regarding the activation of leukemic B cells through BCR, it was similarly reduced by both IVIgGMA and IVIgG. None of these IVIg preparations modified spontaneous apoptosis of T or leukemic B cells. However, the addition of IVIgGMA on in vitro cultures decreased the apoptosis of T cells induced by the BCL-2 inhibitor, venetoclax. Importantly, IVIgGMA did not impair venetoclax-induced apoptosis of leukemic B cells. Overall, our results add new data on the effects of different preparations of IVIg in CLL, and show that the IgM/IgA enriched preparation not only affects relevant mechanisms involved in CLL pathogenesis but also has a particular profile of immunomodulatory effects on T cells that deserves further investigation.

## Introduction

Chronic lymphocytic leukemia (CLL) is the commonest leukemia among adults in western countries. CLL patients have inherent immune defects affecting both cellular and humoral immunity, a condition that is often exacerbated by anti-leukemic therapies. Not surprisingly severe infections are a major cause of morbidity and mortality in patients with CLL^1^. Hypogammaglobulinemia is the most predominant inherent immune defect in CLL, and immunoglobulin replacement therapy (IgRT) is an alternative for patients with hypogammaglobulinemia and recurrent bacterial infections^1^. Although immunoglobulin administrated either intravenously (IVIg) or subcutaneously (SCIg) significantly decreases the rate of bacterial infections of CLL patients, it has no impact in the incidence of non-bacterial infections or in patient overall survival^2^. Currently Ig preparations used in CLL contain more than 95% IgG and as a result, IgA and IgM deficiency persists. An analysis of the factors associated with infections in CLL patients showed a stronger association between major infections and combined antibody deficiency, this is low levels of IgG and IgA or IgM, rather than isolated IgG deficiency^3^. Therefore, one could speculate that the addition of IgA and IgM to Ig preparations might represent an improvement in IgRT in patients with deficiency of all isotypes of Igs, although no studies have addressed this issue yet.

While IVIg preparations were originally developed for IgRT in patients with antibody deficiencies, at higher doses they were found effective as anti-inflammatory therapy in patients with autoimmune or inflammatory diseases^4^. Different mechanisms of action responsible for the immunomodulatory capacity of high doses of IVIg have been identified, for example: direct and indirect inhibition of T-cell activation^5^, induction of anergy and impairment of BCR- and TLR-signalling on B cells^6,7^, and inhibition of the mononuclear phagocytic system^8,9^.

The immunomodulatory capacity of Ig preparations on CLL cells was not directly addressed until recently when Spaner, D. et al. showed that a SCIg preparation impaired BCR signaling, activation and cytokine secretion by CLL cells stimulated in vitro^10^. Interestingly, in that report they found that patients receiving IgRT that increases IgG levels over 9 g/L showed evidence of disease control, suggesting that high doses of Ig may have anti-leukemic activity in CLL patients.

Because both, its particular isotype composition and the chemical treatments during manufacturing might affect the immunomodulatory capacity of an IVIg preparation, our aim was to explore in vitro the immunomodulatory capacity of Pentaglobin, an IVIg enriched in IgM/IgA (IVIgGMA) and Vigam, an IVIg preparation with more than 95% of IgG (IVIgG) in CLL. Given the capacity of IVIg to affect T cell compartment and the particular characteristics of T cells from CLL patients^11^, we extended our analysis not only to leukemic B cells but also to T lymphocytes.

## Results

### The in vitro activation of T cells from CLL patients in response to TCR-stimulation is diminished by IVIgG but not IVIgGMA

Several reports have shown that IgG preparations decreased the activation of T cells from healthy subjects in vitro^5,12,13^. In order to evaluate whether IVIgGMA and IVIgG differentially regulate the activation of T cells from CLL patients, PBMC were stimulated in vitro with immobilized anti-CD3 mAb for 24 h, in the presence of IVIgGMA, IVIgG or HSA at equimolar concentration as control. Because previous reports showed that the inhibitory effect of IgG is observed at high concentrations^5,12,13^, we used both IVIg preparations at a final concentration of IgG of 10 mg/mL.

**Figure 1:**
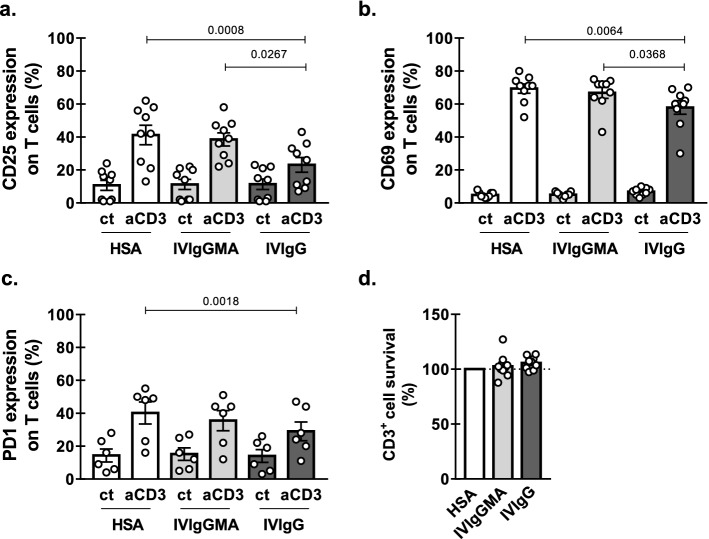
Effect of IVIg preparations on T cell activation. PBMC from CLL patients were cultured in the presence of IVIgGMA, IVIgG (10 mg/mL of IgG) or HSA at equimolar concentration (control), in wells containing immobilized anti-CD3 mAbs (0.5 µg/mL), or the corresponding isotype control as detailed in Materials and Methods. After 24 h CD25 (**a**), CD69 (**b**) and PD1 (**c**) expression was evaluated by flow cytometry on T cell population. Individual values and the mean ± SEM are shown. n = 9 for CD25 and CD69 and n = 6 for PD1. **d.** CD3^+^ cells survival was assessed by flow cytometric alterations of light scattering properties and confirmed by Annexin V staining as detailed in Materials and Methods. n = 9. Individual values are shown. Statistical analysis was performed using Friedman test followed by Dunn’s post-test on **a**, **b** and **c**, and Wilcoxon test on d. Significant *p* values are shown in the graph. *p* < 0.05 was considered significant.

We found that, as already reported for T cells from healthy donors, IVIgG impaired the up-regulation of the activation markers CD25, CD69 and PD-1, while IVIgGMA did not modify their expression (Fig. 1a–c). When the effects of both preparations were compared, we observed that the up-regulation of CD25 and CD69 was significantly lower in the presence of IVIg than in the presence of IVIgGMA (Fig. 1a, b), while no differences were found for PD-1 (Fig. 1c). The inhibition on T cell-activation mediated by IVIgG was dose-dependent showing to be statistically significant at 10 and 1 mg/mL but not at lower doses as previously described for T cells from healthy donors^5^ (see Supplementary Fig. S1 online). Moreover, as shown in Fig. 1d, none of the IVIg preparations affected the viability of CD3^+^ cells of CLL patients.

### The proliferation of T cells from CLL patients in response to TCR-stimulation or IL-15 is differentially affected by the two IVIg preparations

**Figure 2:**
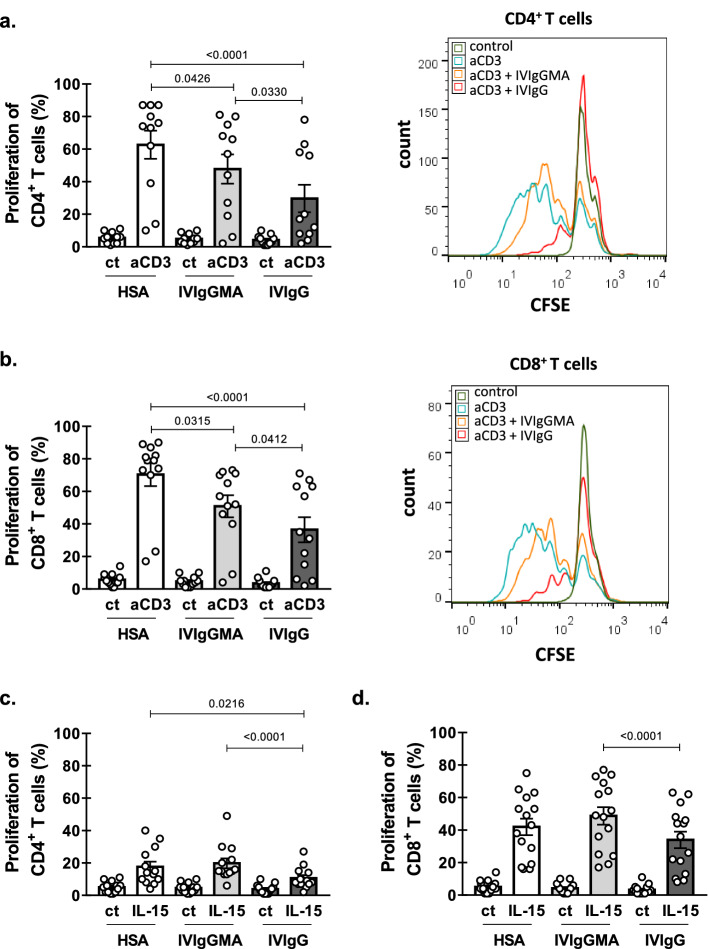
Effect of IVIg preparations on T cell proliferation. PBMC from CLL patients were labeled with CFSE and then cultured, in the presence of IVIgGMA, IVIgG (10 mg/mL of IgG) or HSA, in wells containing immobilized anti-CD3 mAbs (0.5 µg/mL) or the corresponding isotype control or IL-15 (20 ng/mL) as detailed in Materials and Methods. After 5 days, cells were collected, stained with specific mAb for CD4 and CD8 and proliferation was evaluated by flow cytometry. CFSE^low^ cells were those that proliferated. (**a**, **b**) The figures show the percentage of CD4^+^ (**a**) and CD8^+^ (**b**) T cells that proliferated in response to TCR/CD3 stimulation. n = 11 for CD4^+^ T cells and n = 12 for CD8^+^ T cells. Individual values and the mean ± SEM are shown. Friedman test followed by Dunn’s post-test. Representative histograms are shown. (**c-d**) The figures show CD4^+^ (**c**) and CD8^+^ (**d**) cells that proliferated in response to IL-15. n = 13 for CD4^+^ T cells and n = 15 for CD8^+^ T cells. Individual values and the mean ± SEM are shown. Statistical analysis was performed using Friedman test followed by Dunn’s post-test. Significant *p* values are shown in the graph. *p* < 0.05 was considered significant.

Then we evaluated the effect of the different IVIg preparations on T cell proliferation in response to TCR-stimulation and also in response to IL-15, a cytokine involved in homeostatic proliferation of memory T cells. To that aim CFSE-stained PBMC from CLL patients were cultured with immobilized anti-CD3 mAb or IL-15 in the presence of IVIgG, IVIgGMA or HSA. As shown in Fig. 2a, b we found that, contrary to what happened with early activation markers, both IVIg preparations were able to impair T cell proliferation when cells were stimulated through the TCR. This was observed both on CD4^+^ (Fig. 2a) and CD8^+^ T cells (Fig. 2b). Nevertheless, proliferation of T cells in response to TCR-stimulation was significantly lower in the presence of IVIgG than in the presence of IVIgGMA (Fig. 2a, b). T cell proliferation in response to IL-15 was impaired in CD4^+^, but not in CD8^+^ T cells, only by IVIgG preparation (Fig. 2c, d). Again, the proliferation in response to IL-15 was lower in the presence of IVIgG than in the presence of IVIgGMA (Fig. 2c, d). Same results were found when the proliferation of CD8^+^ T cells in response to IL-2 was evaluated (see Supplementary Fig. S2 online).

As shown in the Supplementary Fig. S3 online, the inhibitory effect of the IVIg preparations on the proliferation of T cells from CLL patients was due to a direct effect on this cell population, given that same results were obtained with purified T cells (> 95% T cells).

### Both preparations of IVIg impaired B cell activation in response to BCR crosslinking

**Figure 3:**
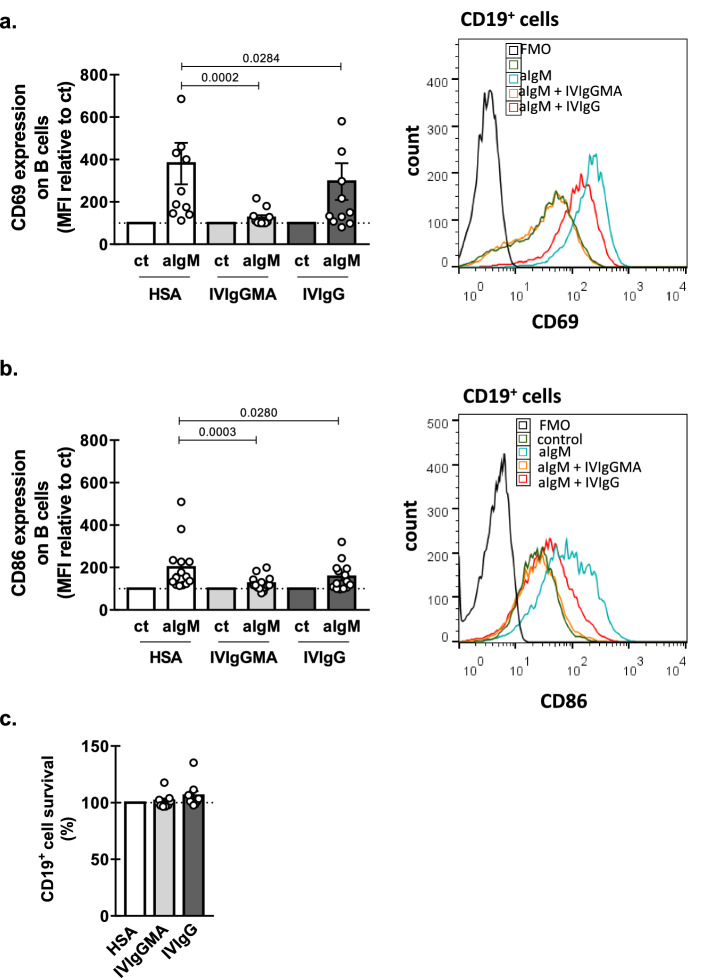
Effect of IVIg preparations on B cell activation. PBMC from CLL patients were cultured, in the presence of IVIgGMA, IVIgG (10 mg/mL of IgG) or HSA, in wells containing immobilized anti-IgM mAbs (25 µg/mL) or the corresponding isotype control, at 37 °C for 24 h as detailed in Materials and Methods. Then CD69 and CD86 expression was analyzed by flow cytometry. (**a, b**) The figures show the expression of the B cells activation markers CD69 (**a**) and CD86 (**b**) on leukemic B cells. Results are shown as the mean fluorescence intensity (MFI) of the activated condition relative to each control condition. n = 10 for CD69 and n = 14 for CD86. Individual values and the mean ± SEM are shown. Representative histograms are shown. (**c**) CD19^+^ cells survival was assessed by flow cytometric alterations of light scattering properties and confirmed by Annexin V staining. n = 9. Individual values are shown. Statistical analysis was performed using Friedman test followed by Dunn’s post-test on (**a**, **b**), and Wilcoxon test on (**c**). Significant *p* values are shown in the graph. *p* < 0.05 was considered significant.

**Figure 4:**
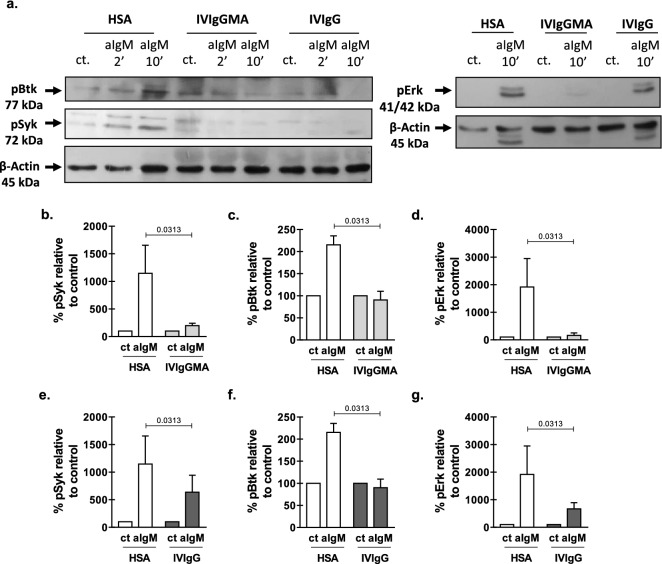
Effect of IVIg preparations on BCR signaling pathway. Purified B-CLL cells (1 × 10^6^ cells) were incubated with IVIgGMA, IVIgG (10 mg/mL of IgG) or HSA and anti-IgM (25 µg/mL) or the corresponding isotype control for 2 or 10 min. Then, cells were lysed and whole cell extracts were prepared as described in Material and Methods. Proteins were separated on a standard 10% SDS-PAGE and transferred to a PVDF membrane. Membranes were probed with primary antibodies for phospho-Syk (pSyk), phospho-Btk (pBtk), phospho-Erk1/2 (pErk) and β-actin, followed by the corresponding secondary antibody as described in Material and methods. Specific bands were visualized by enhanced chemiluminiscence (ECL) method. (**a**) Western blot analysis of pBtk, pSyk and pErk1/2 protein levels. β-actin was included as loading control. Cropped western blot images are shown, full-length blots are presented in Supplementary Fig. S7 and S8 online. Black arrow indicates the specific band. (**b–g**) Quantitative densitometry protein expression relative to β-actin (loading control) is shown. Results are shown as the % of protein expression in activated condition relative to each control condition. The mean ± SEM is shown. Statistical analysis was performed using the Wilcoxon test. n = 5, *p* < 0.05 was considered significant.

As mentioned before, it was recently reported that a SCIg preparation impaired CLL cell activation when stimulated through the BCR in vitro^10^. We asked if the IVIg preparations evaluated herein were also able to decrease leukemic B cell activation. To that aim, PBMC from CLL patients were activated with immobilized anti-IgM mAb to induce BCR crosslinking, in the presence of IVIgGMA, IVIgG or HSA, and after 24 h the expression of the activation markers CD69 and CD86 was assessed. As shown in Fig. 3a, b, both preparations decreased the upregulation of CD69 and CD86, without affecting CD19^+^ cell viability (Fig. 3c). In this case, the inhibitory effect of both preparations was not statistically significant different. The inhibitory effect did not depend on the presence of accessory cells given that similar results were observed with purified leukemic B cells (see Supplementary Fig. S4 online). Moreover, the inhibition on the up-regulation of the activation markers was accompanied with a decrease in the signaling pathway downstream the BCR as shown by a reduced phosphorylation of key molecules such as Syk, Btk and Erk 1/2 (Fig. 4).

The up-regulation of the activation markers CD69 and CD86 in response to BCR cross-linking was significantly reduced by IVIgGMA at 1 and 10 mg/mL or IVIgG at 10 mg/mL, while both preparations at 0.1 mg/mL had no effect (see Supplementary Fig. S5 online). Moreover, the inhibition exerted by both preparations at 10 mg/mL on CLL cell activation seems not to be a general effect but rather specific to particular signalling pathways, given that the inhibition was not observed on CXCL12 or CpG-activated CLL cells (see Supplementary Fig. S6 online).

### IVIgGMA reduced T cell, but not CLL cell, apoptosis induced by venetoclax

**Figure 5:**
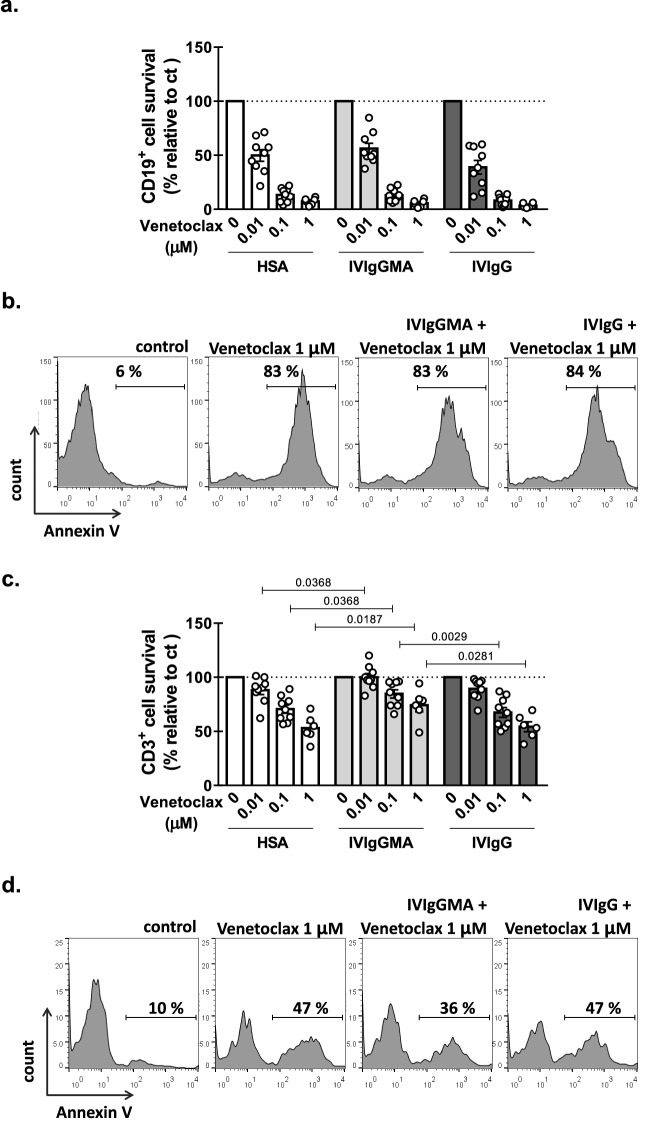
Effect of IVIg preparations on T and B cell apoptosis induced by the BCL-2 inhibitor, venetoclax. PBMC from CLL patients were cultured with IVIgGMA, IVIgG (10 mg/mL of IgG) or HSA. Then, different doses of venetoclax or DMSO (ct) were added to cultures. After 24 h of culture, cell viability was assessed by flow cytometric alterations of light scattering properties and confirmed by staining with Annexin V. (**a**) Percentage of CD19^+^ viable cells. Results are shown as the percentage of viable cells in each condition relative to its control condition. Individual values and the mean ± SEM are shown. (**b**) Representative histograms of Annexin V staining in CD19^+^ cell population are shown. (**c**) Percentage of CD3^+^ viable cell. Results are shown as the percentage of viable cells in each condition relative to its control condition. Individual values and the mean ± SEM are shown. (**d**) Representative histograms of Annexin V staining in CD3^+^ cell population are shown. Statistical analysis was performed using Friedman test followed by Dunn’s post-test. Significant *p* values are shown in the graph. n = 9, *p* < 0.05 was considered significant.

Although we observed that IVIg preparations did not affect the spontaneous apoptosis of leukemic cells (Fig. 1d, 3c), we asked whether these preparations might affect the apoptosis induced by the BCL-2 inhibitor, venetoclax, currently employed in CLL treatment. To that aim PBMC from CLL patients were cultured with clinically relevant doses of venetoclax in the presence of IVIgGMA, IVIgG or HSA. We found that none of the IVIg preparations affect CLL cell apoptosis induced by venetoclax (Fig. 5a, b). Given that we have previously reported that venetoclax induces the apoptosis of T cells from CLL patients^14^, we also evaluated the effect of IVIg preparations on this cell population. Interestingly we found that IVIgGMA reduced T cell apoptosis induced by venetoclax while IVIgG did not (Fig. 5c, d).

## Discussion

The potential benefit of IVIg preparations as an immune-modulator emerged when the infusion of high doses of IVIg in a patient with antibody deficiency and autoimmune thrombocytopenia results in the restoration of the platelets levels to its normal range^15^. Since that initial observation, the use of Igs preparations in the treatment of autoimmune and inflammatory conditions has significantly increased. The study of the mechanisms behind this immunomodulatory effect has expanded as well, and several, not mutually excluding mechanisms have been proposed, including the inhibition of T and B cell activation^4,16^. In CLL, the immunomodulatory capacity of Ig preparations was first suggested in a study from Besa, et al. when they observed a decrease in leukemic cell counts in CLL patients treated with IVIg for recurrent infections and/or autoimmune complications^17^. This observation was recently supported by the work of Spaner, et al. who showed an association between IgG levels above 8–9 mg/mL and a benign course of the disease^10^. They also reported that a SCIg preparation has inhibitory effects on leukemic cells in vitro.

Here we show that two IVIg preparations, one with mainly IgG, and the other enriched with IgA and IgM, interfere with the BCR signalling pathway, decreasing the phosphorylation of early signalling molecules downstream the BCR, as Syk and Btk, and the activation of leukemic cells in response to BCR-stimulation in vitro. Considering the central role of BCR-signalling on CLL pathogenesis, the capacity to inhibit leukemic cell activation adds an attractive immunomodulatory effect to IVIg preparations for CLL patients. The inhibitory effect of both IVIg preparations on BCR-activated leukemic cells did not depend on the presence of accessory cells, since the inhibitory effect was observed either using PBMC or purified leukemic cells from CLL patients. Although we do not know the mechanism by which these preparations impair BCR signalling on leukemic cells, we do know that CLL cells express both FcɣRIIb and CD22^18^, which are receptors that negatively modulate BCR-mediated activation on B cells by interacting with IgG. In that regard, others have shown that blocking FcɣRIIb receptors on CLL cells abrogates IgG mediated inhibition of the activation through the BCR^10^. Also, sialylated IgG present in these preparations might bind CD22 and diminish BCR activation as reported for normal B cells^19^.

We also found that IVIgG impaired the activation of T cells from CLL patients in response to TCR-stimulation, and also in response to other two soluble factors, IL-2 and IL-15, a cytokine involved in homeostatic proliferation of memory T cells. Interestingly, we found that the IVIgGMA preparation had no effect on the upregulation of early activation markers on TCR-stimulated T cells and on the proliferation in response to IL15 or IL-2, while it was able to decrease the proliferation in response to TCR-stimulation. Remarkably IVIgGMA showed a significantly lower inhibitory effect on T cells compared to the IVIgG preparation. The IVIgGMA Pentaglobin, differs from other IVIg preparations, not only in its particular isotype composition (76% IgG, 12% IgM and 12% IgA), but also in its manufacturing process. In order to induce virus inactivation, this IVIgGMA is treated with β-propiolactone, a treatment that also modifies amino-acid residues in the Fc domain of the IgG affecting its binding capacity to monocytes through FcɣR and also its complement fixation capacity^20^. Similar to what we have observed, others have reported that an anti-CMV hyperimmunoglobulin preparation treated with β-propiolactone was less effective in suppressing human T-cell activation in vitro compared to the same Ig preparation without this treatment. Thus, it is possible that β-propiolactone treatment is involved in the lower inhibitory capacity of the IVIgGMA preparation, although we cannot rule out the possibility that the different isotype composition also has consequences on its inhibitory capacity.

When we evaluated the effect of IVIg preparations on the apoptosis induced by venetoclax, we observed that none of the two preparations affected CLL cell viability, while T cell apoptosis induced by venetoclax was significantly lower in the presence of IVIgGMA. Both, leukemic and T cells from CLL patients express high levels of the IgM receptor, the FcµR^21^. Although this receptor was originally described as anti-apoptotic in T cells^22^, convincing studies published later have demonstrated that the receptor has not an anti-apoptotic function per se^23^. Thus the mechanism behind this interesting observation deserves further study.

Our study has two main limitations. First, the fact that both IVIg preparations have differences in their isotype composition along with differences in the production process (mainly β-propiolactone treatment), does not allow us to provide a conclusive explanation for their different effects. Second, patients with unmutated IGVH genes are underrepresented in our cohort (see Supplementary Table S1 online). Considering that this group of patients obtain particular benefit from venetoclax^24^, the observation that pentaglobin prevent T cell apoptosis induced by this drug should be validated with a larger cohort of unmutated patients.

Despite these limitations, the results presented here add new data on the immunomodulatory capacity of IVIg preparations on relevant mechanism involved in CLL pathogenesis.

## Materials and methods

### Reagents and antibodies

RPMI 1640 was purchased from Life Technologies (USA), penicillin and streptomycin from GIBCO (USA) and fetal calf serum from Natocor (Argentina). Bovine serum albumin (BSA) was obtained from Wiener Laboratorios (Argentine).

FITC-, PE- or PercP conjugated mAbs specific for CD3 (HIT3a), CD4 (OKT4), CD5 (L17F12), CD8 (HIT8a) and CD25 (M-A251) were obtained from BioLegend (USA). FITC- and PE-conjugated mAbs specific for CD69 (FN50), CD86 (2331) and Annexin-V FITC from BD Bioscience, Pharmingen (USA). PC5-conjugated mAb specific for CD19 (J3-119) and purified anti-CD3 (UCHT1) were obtained from Beckman Coulter (USA) and anti-human-IgM mAb from Jackson ImmunoResearch (USA).

For western blot, polyclonal antibodies (pAb) specific for phospho-ZAP-70 (Tyr319)/SYK (Tyr352), pAb specific for phospho-BTK (Tyr223) and mAb for β-actin (8H10D10) were obtained from Cell Signaling (USA). mAb specific for phospho-ERK1/2 (Thr202/Tyr204) was obtained from BioLegend. The HRP-conjugated mAb for mouse IgG was purchased from Sigma-Aldrich (USA) and the HRP-conjugated mAb for rabbit IgG from Jackson ImmunoResearch, Inc.

Carboxyfluorescein succinimidyl ester (CFSE) was obtained from Invitrogen (Argentine), dimethyl sulfoxide (DMSO) from Sigma-Aldrich (USA) and Venetoclax (ABT-199) from MedKoo Biosciences (USA).

CD3 and B-CLL human Microbeads isolation kits were obtained from Miltenyi biotec (Germany).

CpG was obtained from Integrated DNA technologies (USA) and CXCL12 from PeproTech (Mexico). IL-15 and IL-2 were obtained from BioLegend.

Pentaglobin (IVIgGMA) from Biotest (Germany) was gently provided by Microsules Agentina S.A. Vigam Liquid (IVIgG) was obtained from Bio Products Laboratories (UK) and human serum albumin (HSA) from Universidad Nacional de Córdoba (Argentine).

HSA at equimolar concentrations is commonly used as protein control on in vitro experiments with IVIg^5,12,25,26^ mainly to rule out the possibility that the observed effect is not solely caused by the addition of protein to the cell cultures.

### CLL patient samples and cell separation procedures

The study was approved by the local ethics committee from Academia Nacional de Medicina, Buenos Aires, Argentina, according to the institutional guidelines (Approval number 15/20/CEIANM). Peripheral blood samples were obtained from CLL patients after signed informed consent. The study was conducted according to the principles of the Declaration of Helsinki.

CLL was diagnosed according to standard clinical and laboratory criteria. At the time of the analysis patients were free from clinically relevant infectious complications and were either untreated or had not received treatment for a period of at least 6 months before investigation. Clinical characteristics of CLL patients included in the study are shown in Supplementary Table S1.

Peripheral blood samples were obtained from CLL patients and peripheral blood mononuclear cells (PBMC) were isolated as previously described^14^.

T cells from CLL patients were purified by positive selection with the anti-CD3 Microbead isolation kit (purity obtained > 95%). Leukemic B cells from CLL patients were obtained by negative selection with the anti-B-CLL Microbead isolation kit (purity obtained > 98%). Magnetic separation was performed according to manufacturer’s instructions.

### T cell cultures

For T cell activation, PBMC (3 × 10^5^ cells/150 µL RPMI 10% FCS) were pre-incubated for 30 min at 37 °C with IVIgGMA, IVIgG (0.1–1–10 mg/mL of IgG) or HSA at equimolar concentration (control) and then cultured on a 96-well culture plate containing immobilized anti-CD3 mAbs (0.5 µg/mL) or the corresponding isotype control for 24 h at 37 °C. Then, cells were stained with mAb for CD4, CD8, CD25, CD69 and PD1 and evaluated by flow cytometry as detailed in the Flow cytometry section. The presence of HSA did not affect T cell activation (not shown).

T cell proliferation was evaluated using the CFSE dilution assay. PBMC or purified T cells, both from CLL patients (3 × 10^5^ cells/150 µL RPMI 10% FCS) were labelled with CFSE (1 µM) and then pre-treated for 30 min at 37 °C with IVIgGMA, IVIgG (10 mg/mL of IgG) or HSA. Then, cells were cultured on a 96-well culture plate containing immobilized anti-CD3 mAbs (0.5 µg/mL) or the corresponding isotype control or IL-15 (20 ng/mL) or IL-2 (600 U/mL) for 5 days at 37 °C. Cells were then collected, stained with mAb for CD4 and CD8 and proliferation evaluated by flow cytometry. Percentage of proliferation was determinate as the % of T cells with low stain of CFSE.

### B cell cultures

PBMC or purified B-CLL cells (3 × 10^5^ cells/150 µL RPMI 10% FCS) from CLL patients were pre-incubated with immobilized anti-IgM mAbs (25 µg/mL) or the corresponding isotype control for 30 min at 4 °C. Then, IVIgGMA, IVIgG (0.1–1–10 mg/mL of IgG) or HSA was added, and cells were cultured for 24 h at 37 °C. The expression of CD19, CD86 and CD69 was evaluated by flow cytometry. The presence of HSA did not affect B cell activation (not shown).

PBMC were pre-treated for 30 min at 37 °C with IVIgGMA, IVIgG or HSA and then CpG (1 µM) or CXCL12 (500 ng/mL) were added to cultures. After 24 h, B cell activation was evaluated by flow cytometry.

### Venetoclax-induced apoptosis cultures

PBMC from CLL patients (3 × 10^5^ cells/150 µL RPMI 10% FCS) were pre-treated in a 96-well plate for 30 min at 37 °C with IVIgGMA, IVIgG (10 mg/mL of IgG) or HSA. Then, different doses of Venetoclax (0.01–0.1–1 µM) or DMSO (drug vehicle) were added to cultures. After 24 h cell viability was assessed by flow cytometric alterations of light scattering properties and confirmed by staining with CD19, CD3 and Annexin V.

### Flow cytometry

For surface staining, cells were incubated with the corresponding antibodies, anti-CD3, CD4, CD5, CD8, CD19, CD25, CD69 and CD86, for 30 min at 4 °C. The staining was performed in phosphate-buffered saline-0.5% BSA. Cells were then washed and fixed with paraformaldehyde 1%. Gating of populations positive for any particular marker was based on fluorescence minus one (FMO) control of each activation marker. FMO was not modified by activation or IVIg treatment (data not shown).

Cell viability was determined by Annexin V staining performed on binding buffer. After cell surface staining, cells were washed once with binding buffer and then stained with Annexin V for 20 min at room temperature. Samples were then acquired in the flow cytometry.

Cell viability was also determined by flow cytometry by evaluating flow cytometric alteration of light scattering properties as previously described^14,27^. Briefly, apoptotic lymphocytes can be distinguished from viable lymphocytes by flow cytometry by their differences in cell morphology^28^. Apoptotic cells show a reduction in cell size (lower FSC), and as a result of chromatin condensation, nucleus fragmentation and cytoplasmic protein cross-linking, in the late stages of the apoptosis process the scattering of light in SSC is decreases (lower SSC)^29^. The analysis was performed evaluating the FSC-H vs SSC-H parameters, both on a linear scale.

Samples were acquired with a BD FACSCalibur (BD Biosciences) and data were analyzed with FlowJo 10 software (FlowJo, USA).

### Western blot

Purified B-CLL cells (2 × 10^6^ cells) were activated with anti-IgM (25 µg/mL) or the corresponding isotype control at 37 °C, in the presence of IVIgGMA, IVIgG (10 mg/mL of IgG) or HSA. After 2 or 10 min, the reaction was stopped with cold saline solution. Whole-cell lysates were prepared using 75 µL of RIPA buffer containing protease inhibitors (Thermo Fischer Scientific, #78440). Lysates were vortex and incubated on ice twice, and then after centrifugation, supernatants were transferred to a new tube. 25 µL of loading buffer 4× containing β-mercaptoethanol was added and then samples were incubated for 5 min at 95 °C. 50 µL of protein extracts were separated on a standard 10% SDS-PAGE and transferred to PVDF membranes (GE HealthCare Science, #GE1060023). Membranes were then blocked with a PBST solution containing 5% non-fat dry milk for 2 h at room temperature. Then, PVDF membranes were cut to perform the incubation of the different sections of the same membrane with different primary antibodies, as previously reported^30^. Membranes were incubated with primary antibodies over night at 4 °C as follows: membranes with molecular weight marker between 100 and 50 kDa were probed with anti-phospho-SYK and anti-phospho-BTK and membranes with molecular weight marker below 50 kDa were probed with anti-phospho-ERK1/2 and anti-β-actin. Membranes were then incubated with the corresponding secondary antibody, HRP-conjugated anti-rabbit or anti-mouse IgG mAb, for 1 h at room temperature. Specific bands were visualized by enhanced chemiluminiscence (ECL) method. The expression of β-actin was used as a loading control to normalize the protein levels detected in each lane of the same gel. The molecular weight marker was the Precision Plus Protein™ All Blue Prestained Protein Standards (10–250 kDa) from BioRad (#1610373). Densitometric measurements of specific bands were determinate by using ImageJ software (NIH).

### Statistical analysis

Statistical significance was determined using non-parametric tests: Wilcoxon matched-pairs signed rank test to compare between two paired groups and Friedman followed by the Dunn’s post-test to compare three or more groups. Two-tailed tests were used and *p* < 0.05 was considered statistically significant. The corresponding *p* value is indicated. Data were analysed using the GraphPad Prism software version 7.

## Supplementary Information

**Supplementary Information**.
