# Supplementary material for: Immunomodulatory effects of different intravenous immunoglobulin preparations in chronic lymphocytic leukemia
Source: Sci Rep. 2021 Jun 21;11:12926. doi: 10.1038/s41598-021-92412-8 (PMC8217488; doi:10.1038/s41598-021-92412-8)
Supplement: Supplementary file 1 — Supplementary Information. [file 41598_2021_92412_MOESM1_ESM.pdf]

Immunomodulatory effects of different intravenous immunoglobulin preparations in chronic lymphocytic leukemia

Ana Colado[1], Esteban Enrique Elías[1], Valeria Judith Sarapura Martínez[1], Gregorio Cordini[1], Pablo Morande[1,2], Fernando Bezares[3], Mirta Giordano[1,4], Romina Gamberale[1,4] and Mercedes Borge* [1,4].

[1] Laboratorio de Inmunología Oncológica, Instituto de Medicina Experimental (IMEX)-CONICET-Academia Nacional de Medicina (ANM), CABA, Argentina.

[2] Tumor Stroma Interactions, Department of Oncology, Luxembourg Institute of Health, L-1526 Luxembourg, Luxembourg.

[3] Hospital General de Agudos Dr. Teodoro Álvarez, CABA, Argentina.

[4] Departamento de Microbiología, Parasitología e Inmunología, Facultad de Medicina, Universidad de Buenos Aires, CABA, Argentina.

*** Corresponding Author:**
Mercedes Borge
email: mborge@fmed.uba.ar
ORCID: 0000-0002-1256-2364
Laboratorio de Inmunología Oncológica, IMEX-CONICET-ANM. Pacheco de Melo 3081 (1425), CABA, Argentina.
Phone number: +54 11 4805 3411

FAX number: + 54 11 4803 9475

Supplementary figure S1

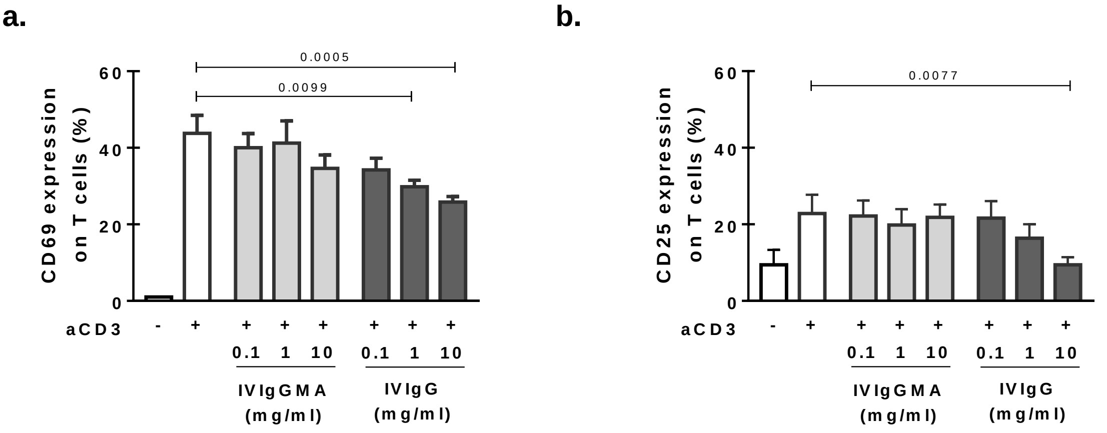

Fig S1. Effect of different doses of IVIg preparations on T cell activation. PBMC ($3x10^5$ cells/150 µl RPMI 10% FCS) were activated for 24 hours at 37°C with immobilized anti-CD3 mAbs (0.5 µg/ml) or the corresponding isotype in the presence of IVIgGMA, IVIgG (0.1-1-10 mg/ml of IgG) or HSA. Then, cells were stained with mAb for CD4, CD8 and CD69 **(a)** or CD25 **(b)** and T cell activation was evaluated by flow cytometry. Results are shown as the % of CD69 or CD25 expression on T cell population. The mean ± SEM is shown, n=4. Statistical analyses were performed using Friedman test followed by Dunn's post-test. $p < 0.05$ was considered significant

Supplementary figure S2

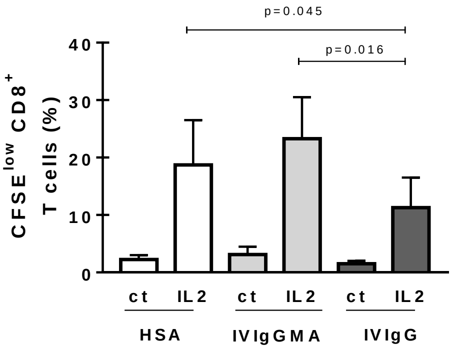

Fig S2. Effect of IVIg preparations on IL-2-induced T cell proliferation. PBMC ($3x10^5$ cells/150 µl RPMI 10% FCS) were labelled with CFSE (1µM) and then cultured for 30 minutes at 37°C with IVIgGMA, IVIgG (10 mg/ml of IgG) or HSA. Then, cells were transferred to wells containing IL-2 (600 U/ml) and cultured at 37°C for 5 days. Cells were then collected, stained with mAb for CD8 and the proliferation was evaluated by flow cytometry. The percentage of proliferation was determinate as the % of T $CD8^+$ cells with low stain of CFSE. The mean ± SEM is shown. Statistical analysis was performed using Friedman test followed by Dunn's post-test. n=8, $p < 0.05$ was considered significant

Supplementary figure S3

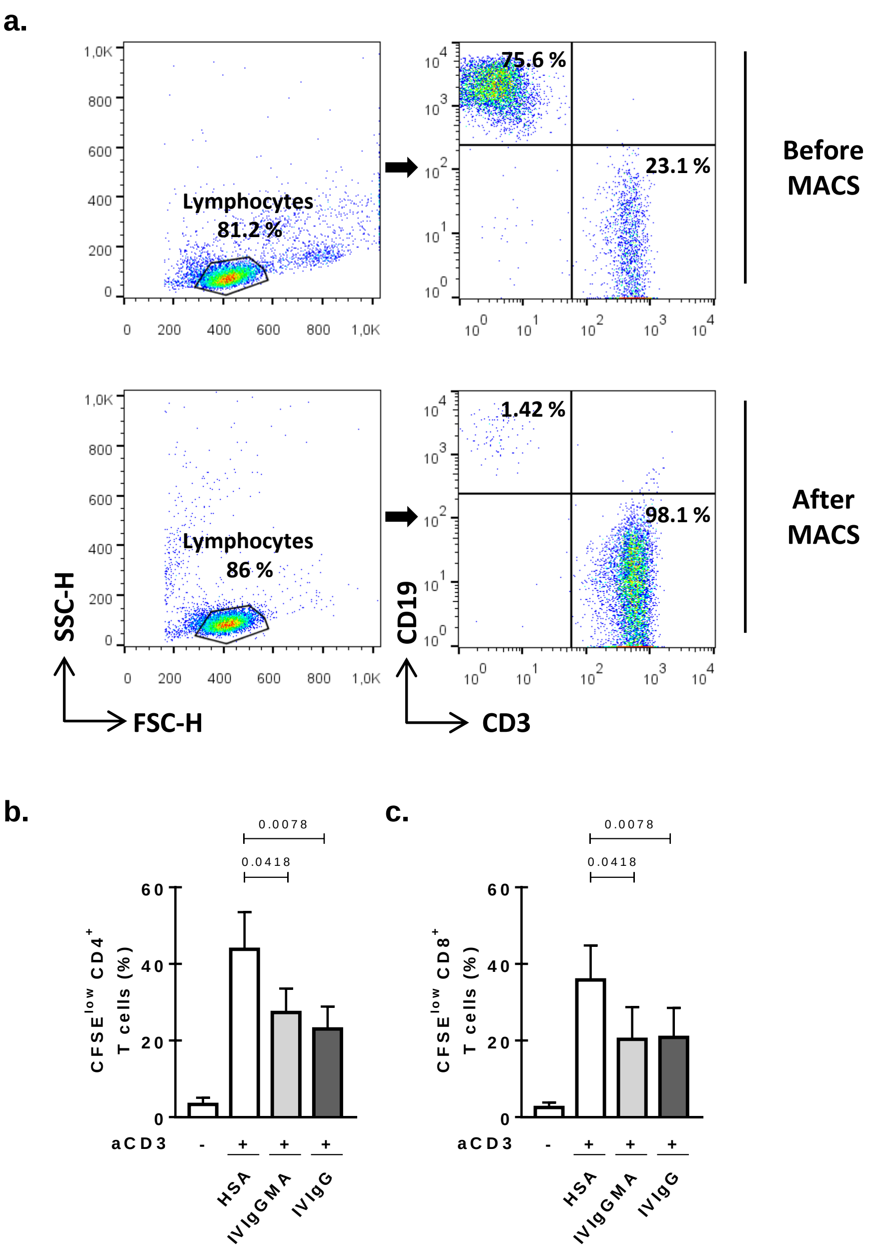

Fig S3. Effect of IVIg preparations on purified T cell proliferation. T cells from CLL patients were purified by positive selection with the anti-CD3 Microbead isolation kit (Miltenyi Biotec). **a.** Representative dot plots showing the % of CD3^{+} cells in the sample, before and after magnetic separation, are shown. **b-c.** Purified T cells (3x10^{5} cells/150 µl RPMI 10% FCS) were labelled with CFSE (1µM) and then cultured on wells containing immobilized anti-CD3 mAbs (0.5 µg/ml) or the corresponding isotype control, in the presence of IVIgGMA, IVIgG (10 mg/ml of IgG) or HSA and cultured at 37°C for 5 days. Cells were then collected, stained with mAb for CD4 and CD8 and the proliferation was evaluated by flow cytometry. The percentage of proliferation was determinate as the % of T CD4^{+} **(b)** or CD8^{+} **(c)** cells with low stain of CFSE. The mean ± SEM is shown. Statistical analysis was performed using Friedman test followed by Dunn’s post-test. n=6, $p < 0.05$ was considered significant

Supplementary figure S4

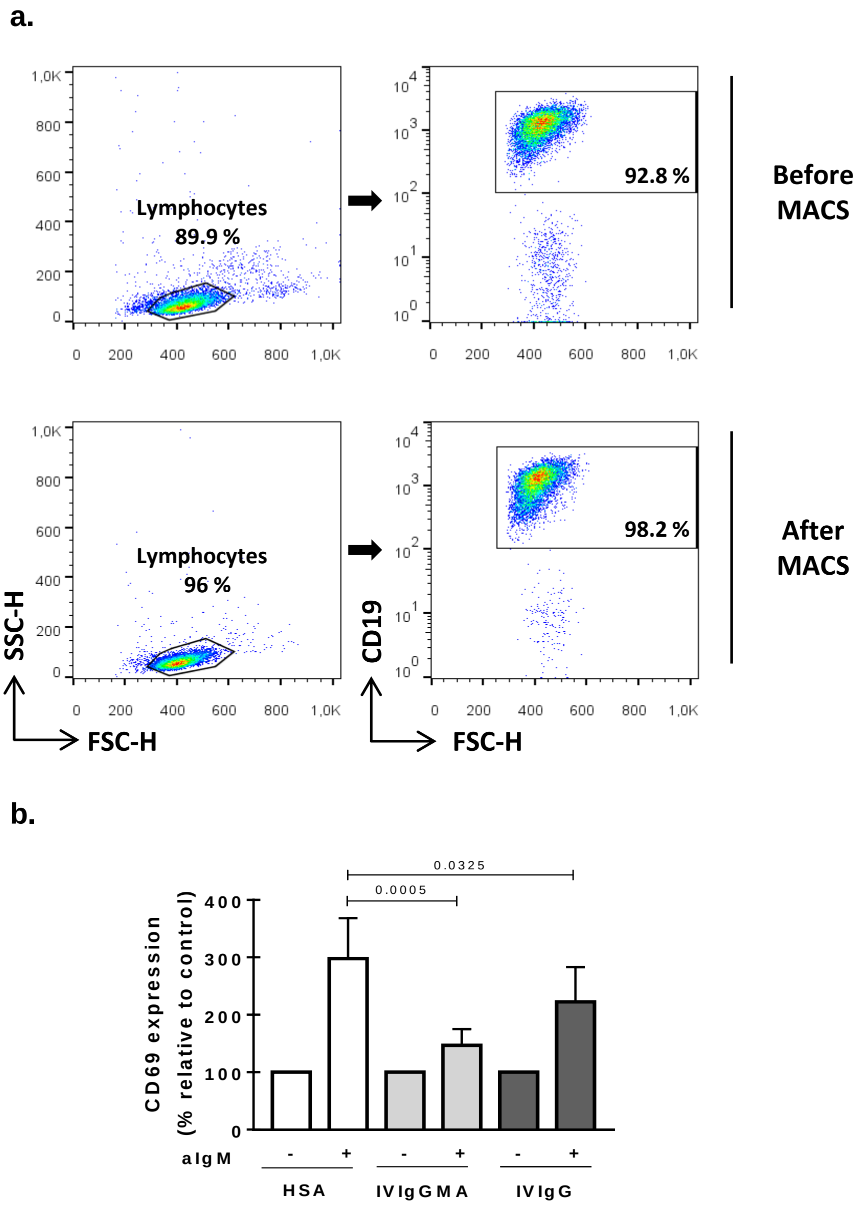

Fig S4. Effect of IVIg preparations on purified B-CLL cell activation. B-CLL cells from CLL patients were obtained by negative selection with the anti-B-CLL Microbead isolation kit (Miltenyi Biotec). **a.** Representative dot plots showing the % of CD19$^+$ cells in the sample, before and after magnetic separation, are shown **b.** B-CLL cells ($3x10^5$ cells/150 µl RPMI 10% FCS) were cultured with anti-IgM mAbs (25 µg/ml) or the corresponding isotype control in the presence of IVIgGMA, IVIgG (10 mg/ml of IgG) or HSA at 37°C for 24 hours. Then, cells were stained with anti-CD19 and CD69 and B cell activation was evaluated by flow cytometry. Results are shown as the mean fluorescence intensity (MFI) of the activated condition relative to each control condition. The mean ± SEM is shown. Statistical analysis was performed using Friedman test followed by Dunn's post-test. n=7, $p < 0.05$ was considered significant

Supplementary figure S5

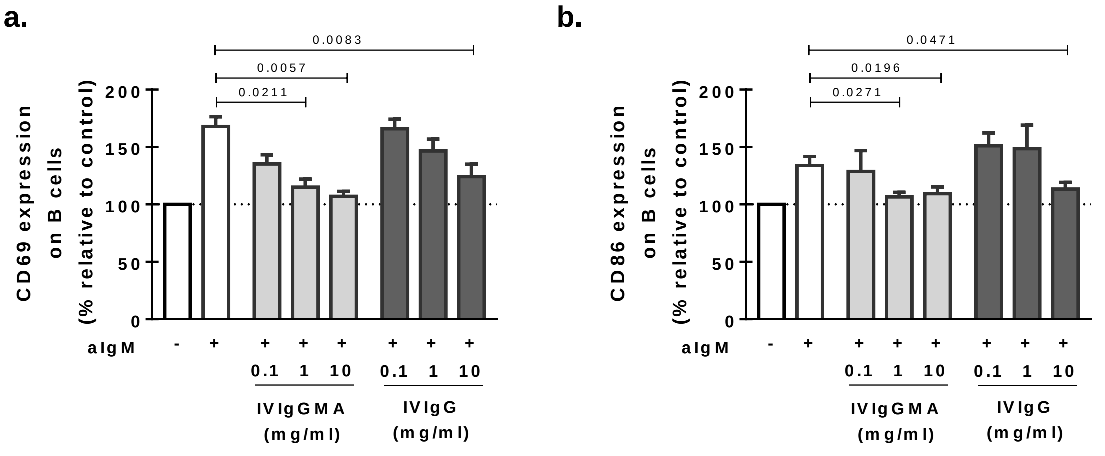

Figure S5. Effect of different doses of IVIg preparations on B cell activation. PBMC ($3x10^5$ cells/150 µl RPMI 10% FCS) were cultured for 24 hours at 37ºC with anti-IgM mAbs (25 µg/ml) in the presence of different concentrations of IVIgGMA or IVIgG (0.1, 1 and 10 mg/ml of IgG), or HSA (at an equimolar concentration of each concentration of IgG). Then, cells were stained with anti-CD19 and CD69 **(a)** or CD86 **(b)** and B cell activation was evaluated by flow cytometry. Results are shown as the mean fluorescence intensity (MFI) of the activated condition relative to the control condition. The mean ± SEM is shown, n=4. Statistical analyses were performed using Friedman test followed by Dunn's post-test. p <0.05 was considered significant

Supplementary figure S6

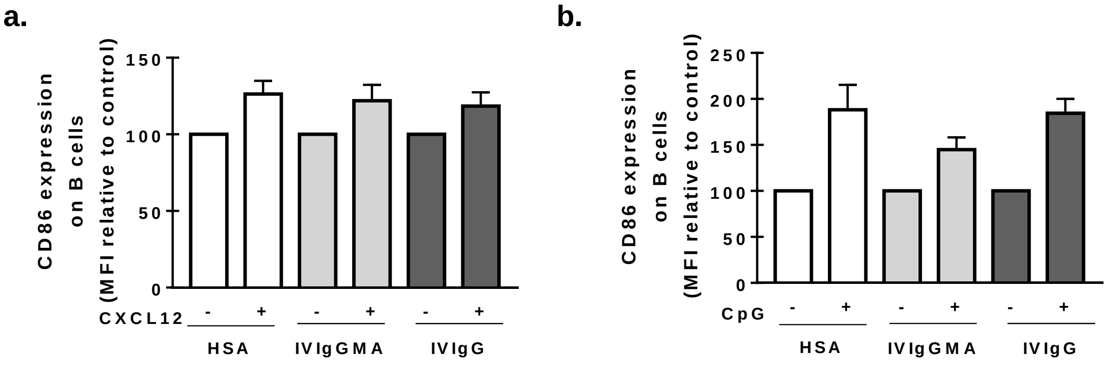

Fig S6. Effect of IVIg preparations on CXCL12- and CpG-induced B cell activation. PBMC from CLL patients ($3x10^5$ cells/150 µl RPMI 10% FCS) were cultured for 24 hours at 37°C with CXCL12 (500 ng/ml) **(a)** or CpG (1 µM) **(b)** in the presence of IVIgGMA, IVIgG (10 mg/ml of IgG) or HSA. Then, cells were stained with anti-CD19 and CD86. B cell activation was evaluated by flow cytometry. Results are shown as the mean fluorescence intensity (MFI) of the activated condition relative to each control condition. The mean ± SEM is shown. n=5 for CXCL12 experiments and n=7 for CpG experiments. Statistical analysis was performed using Friedman test followed by Dunn's post-test and no statistically significant differences were observed between treatment with IVIgG or IVIgGMA vs control

Supplementary figure S7

a.

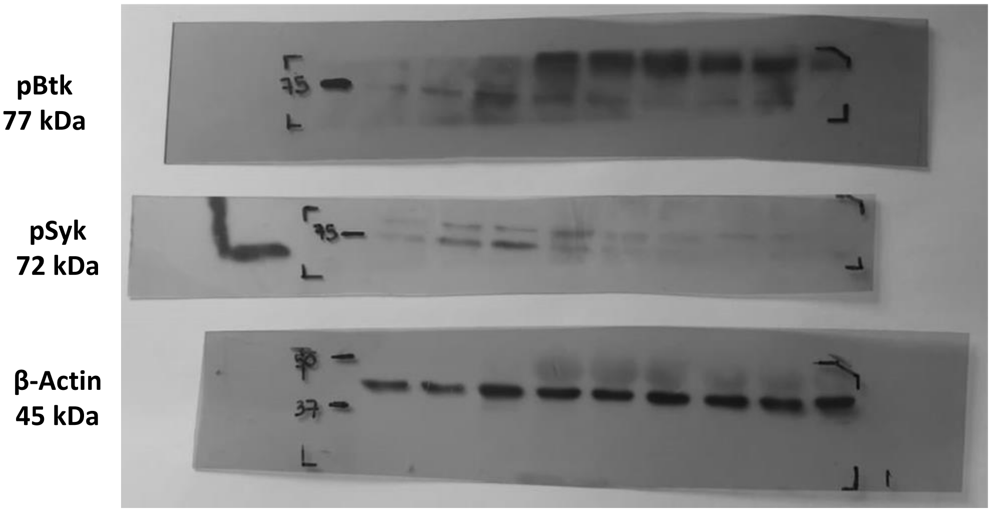

b.

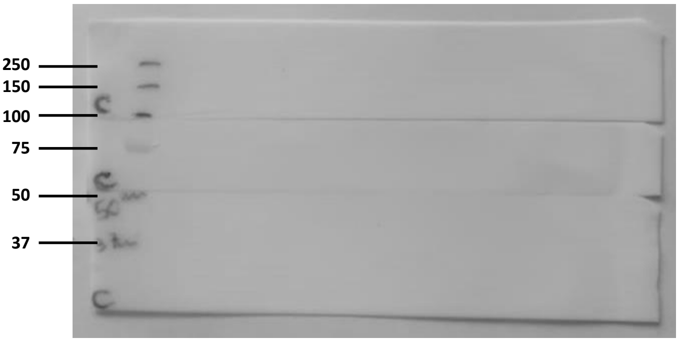

Fig S7. Full-length blots for cropped western blots in Fig 4a. a. Western blot analysis for pBTK (Tyr223), pSYK (Tyr352) and β-actin. β-actin protein levels were used as loading control. From the left: Molecular weight marker, HSA + ct, HSA + aIgM (2'), HSA + aIgM (10'), IVIgGMA + ct, IVIgGMA + aIgM (2'), IVIgGMA + aIgM (10'), IVIgG + ct, IVIgG + aIgM (2'), IVIgG + aIgM (10'). **b.** Full blot image. The following primary antibodies were used: polyclonal antibodies (pAb) specific for phospho-ZAP-70 (Tyr319)/SYK (Tyr352), pAb specific for phospho-BTK (Tyr223) and monoclonal antibodies (mAb) for β-actin (8H10D10) from Cell Signaling (USA). The blot was cut to perform the incubation of the different sections of the same membrane with different primary antibodies as previously reported (Salati, Genovese et al. 2019) and membranes were incubated with primary antibodies over night at 4°C as follows: membranes with molecular weight marker between 100 kDa and 50 kDa were probed with anti-phospho-SYK and anti-phospho-BTK and membranes with molecular weight marker below 50 kDa were probed with anti-phospho-ERK1/2 and anti-β-actin. Membranes were then incubated with the corresponding secondary antibody, HRP-conjugated anti-rabbit or anti-mouse IgG mAb, for 1 hour at room temperature. Specific bands were visualized by enhanced chemiluminiscence (ECL) method. The expression of β-actin was used as a loading control to normalize the protein levels detected in each lane of the same gel. The molecular weight marker was the Precision Plus Protein™ All Blue Prestained Protein Standards (10-250 kDa) from BioRad (#1610373). Densitometric measurements of specific bands were determinate by using ImageJ software (NIH).

Supplementary figure S8

a.

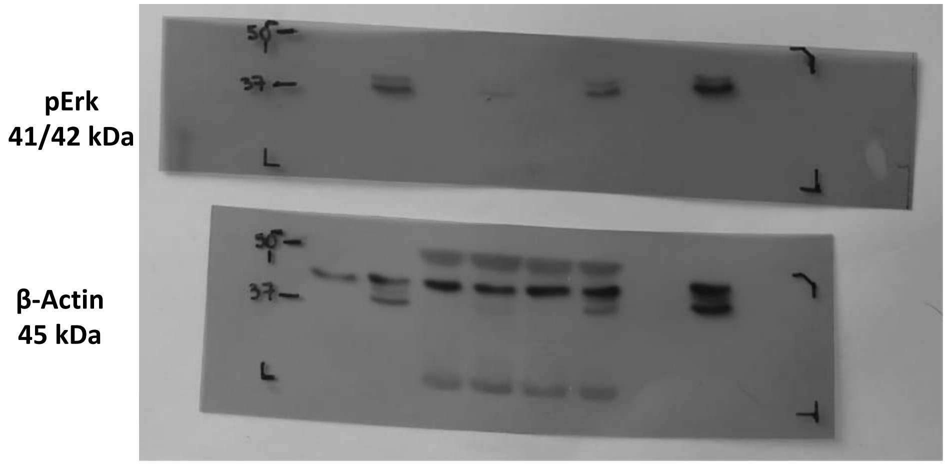

b.

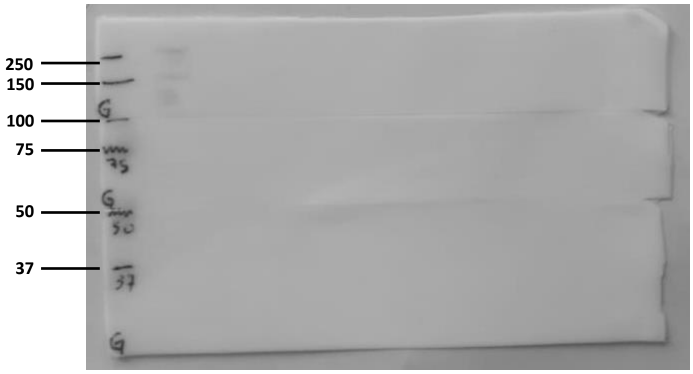

Fig S8. Full-length blots for cropped western blots in Fig 4a. a. Western blot analysis for pErk1/2 and β-actin. β-actin protein levels were used as control. From the left: Molecular weight marker, HSA + ct, HSA + aIgM (10’), IVIgGMA + ct, IVIgGMA + aIgM (10’), IVIgG + ct, IVIgG + aIgM (10’), empty lane, positive control, empty lane. **b.** Full blot image. The following primary antibodies were used: monoclonal antibodies (mAb) for β-actin (8H10D10) from Cell Signaling (USA) and for phospho-ERK1/2 (Thr202/Tyr204) mAb from BioLegend. The blot was cut to perform the incubation of the different sections of the same membrane with different primary antibodies as previously reported (Salati, Genovese et al. 2019) and membranes were incubated with primary antibodies over night at 4°C as follows: membranes with molecular weight marker between 100 kDa and 50 kDa were probed with anti-phospho-SYK and anti-phospho-BTK and membranes with molecular weight marker below 50 kDa were probed with anti-phospho-ERK1/2 and anti-β-actin. Membranes were then incubated with the corresponding secondary antibody, HRP-conjugated anti-rabbit or anti-mouse IgG mAb, for 1 hour at room temperature. Specific bands were visualized by enhanced chemiluminiscence (ECL) method. The expression of β-actin was used as a loading control to normalize the protein levels detected in each lane of the same gel. The molecular weight marker was the Precision Plus Protein™ All Blue Prestained Protein Standards (10-250 kDa) from BioRad (#1610373). Densitometric measurements of specific bands were determinate by using ImageJ software (NIH).

.

Supplementary Table S1

Table S1. Clinical and biological features of CLL patients enrolled in the study.

CLL patient #	Gender	Age (years)	dx year/ sample year	Binet	Leucocytes ($x10^9/L$)	Lymphocytes ($x10^9/L$)	CD19+ (%) *	CD38+ (%) Δ	CD49d+ (%) λ	Hb (g/dl)	Platelets ($x10^3/\mu l$)	LDH (U/L)	β2micro (µg/ml)	IgG (mg/dl)	IgA (mg/dl)	IgM (mg/dl)	IGHV mutational status	TP53 mutation	FISH	Recurrent Infections
1	Male	70	2015/2019	B	105	94.5	90	3	1	13.5	111	178	3.16	510	48	19	M	M	Del17p13	No
2	Male	76	1992/2015	C	45.5	39.1	89	3	67	14	89	390	3.1	499	29	12	M	M	Del17p13<br>Del13q14<br>Del11q23	No
3	Female	84	1996/2019	A	68.1	54.8	92	5	6	12.8	211	171	2.0	700	70	30	M	UM	Del13q14	No
4	Female	70	2004/2017	A	26.4	21.5	90	1	8	12.6	153	396	4.5	522	43	16	M	UM	Neg	Yes
5	Male	70	2016/2017	B	100	92	89	77	44	12.4	175	403	n.d.a	600	56	30	n.d.a	UM	Del17p13	No
6	Male	85	2012/2017	C	650	539	97	10	1	7.8	102	424	n.d.a	590	45	30	M	UM	t12	Yes
7	Male	60	2007/2018	B	26	23.4	89	1	0	14.7	76	334	3.10	723	37	20	M	UM	Del13q14; t12	No
8	Male	59	2013/2017	A	83.1	78.7	76	70	23	11.5	160	250	2.71	650	64	38	M	UM	Del13q14	No
9	Male	56	2009/2015	A	29.1	27	87	1	35	13.8	167	310	2.2	n.d.a	n.d.a	n.d.a	M	n.d.a	n.d.a	n.d.a.
10	Male	54	2013/2015	A	18.7	13.1	71	24	26	12	194	337	1.91	n.d.a	n.d.a	n.d.a	n.d.a	n.d.a	n.d.a	n.d.a
11	Male	77	2006/2017	A	36.5	29.5	82	11	5	13.8	215	344	n.d.a	968	79	106	M	M	Del13q14	No
12	Male	67	n.d.a/2019	B	181	128.5	86	30	97	13.2	358	n.d.a	n.d.a	n.d.a	n.d.a	n.d.a	n.d.a	n.d.a	n.d.a	n.d.a
13	Male	68	2010/2018	A	18.5	14.8	47	5	8	15.6	160	300	1.90	765	65	50	M	UM	Del13q14<br>Normal karyotype	No
14	Male	59	2016/2018	C	34.1	29.2	90	97	99	14	90	374	2.75	306	33	7	M	UM	Neg	No
15	Male	73	n.d.a/2019	n.d.a	26.9	19.1	89	1	1	n.d.a	174	n.d.a	n.d.a	n.d.a	n.d.a	n.d.a	n.d.a	n.d.a	n.d.a	n.d.a
16	Male	64	2001/2017	C	9.9	4.5	69	14	67	11.2	82	454	3.1	409	18	44	UM	UM	Del17p13	Yes
17	Female	55	2013/2018	A	47	39	85	0.5	1	12.7	290	408	1.7	631	102	42	M	UM	Neg	No
18	Female	52	2014/2015	C	56	49.7	89	51	2	14.2	131	520	4.0	529	50	20	UM	n.d.a	Del17p13<br>Del11q23<br>Complex karyotype	Yes
19	Female	76	2001/2017	A	48.5	44.1	93	1	1	14.6	167	270	2.87	630	58	32	n.d.a	UM	Neg	No
20	Female	68	2017/2018	B	89	79	95	89	91	10	86	560	4.0	670	46	25	UM	n.d.a	t12; Del13q14	Yes
21	Female	65	2016/2017	C	426	413	98	0.5	76	8.4	206	409	4.5	900	115	46	UM	UM	Neg	No

Δ Percentage of CD38+ cells in CD19+ lymphocytes. λ Percentage of CD49d+ cells in CD19+ lymphocytes. β2micro, β-2 microglobulin. LDH, lactate dehydrogenase. Hb, hemoglobin. IGHV, immunoglobulin heavy chain variable región. M, mutated. UM, unmutated. Neg, negative; t12, trisomy 12. n.d.a, no data available

Immunoglobulins values shown correspond to values at the time of sample colection. Normal range: IgA 70-400 mg/dl; IgG 700-1600 mg/dl; IgM 40-230 mg/dl. Recurrent infections: ≥4 infections per year.
